# A field test on the effectiveness of male annihilation technique against *Bactrocera dorsalis* (Diptera: Tephritidae) at varying application densities

**DOI:** 10.1371/journal.pone.0213337

**Published:** 2019-03-08

**Authors:** Nicholas C. Manoukis, Roger I. Vargas, Lori Carvalho, Thomas Fezza, Shannon Wilson, Travis Collier, Todd E. Shelly

**Affiliations:** 1 United States Department of Agriculture- Agricultural Research Service, Daniel K. Inouye US Pacific Basin Agricultural Research Center, Hilo, HI, United States of America; 2 United States Department of Agriculture- Animal Plant Health Inspection Service, Waimanalo, HI, United States of America; University of Thessaly School of Agricultural Sciences, GREECE

## Abstract

Male Annihilation Technique (MAT) is a key tool to suppress or eradicate pestiferous tephritid fruit flies for which there exist powerful male lures. In the case of *Bactrocera dorsalis* (Hendel), a highly invasive and destructive species, current implementations of MAT utilize a combination of the male attractant methyl eugenol (ME) and a toxicant such as spinosad (“SPLAT-MAT-ME”) applied at a high density with the goal of attracting and killing an extremely high proportion of males. We conducted direct comparisons of trap captures of marked *B*. *dorsalis* males released under three experimental SPLAT-MAT-ME site densities (110, 220, and 440 per km^2^) near Hilo, Hawaii using both fresh and aged traps to evaluate the effectiveness of varying densities and how weathering of the SPLAT-MAT-ME formulation influenced any density effects observed. Counterintuitively, we observed decreasing effectiveness (percent kill) with increasing application density. We also estimated slightly higher average kill for any given density for weathered grids compared with fresh. Spatial analysis of the recapture patterns of the first trap service per replicate x treatment reveals similar positional effects for all grid densities despite differences in overall percent kill. This study suggests that benefits for control and eradication programs would result from reducing the application density of MAT against *B*. *dorsalis* through reduced material use, labor costs, and higher effectiveness. Additional research in areas where MAT programs are currently undertaken would be helpful to corroborate this study’s findings.

## Introduction

Males of many species of true fruit flies (Diptera: Tephritidae) are attracted to a small set of plant-derived secondary compounds termed male lures [1–3]. In the subfamily Dacini, males of the genera *Bactrocera* Macquart and *Zeugodacus* Hendel may be categorized broadly as responding either to methyl eugenol (ME) or raspberry ketone (RK) or its hydrolyzed form cue-lure (CL) [4,5] (but see [6,7] for recent data challenging this classification). It is widely believed [2], and limited field data support [8–10], that ME is a more powerful attractant than RK/CL. Why males respond to these lures was unknown until recently, but numerous studies (e.g., [11,12]) have now demonstrated that feeding on lures enhances male sexual behavior and signaling, which results in increased mating success.

The natural association between males and lures has been coopted as a key tool in controlling tephritid pest species, many of which, because of their broad host range, high vagility, and invasive capability, pose serious global threats to many important agricultural crops (e.g., [13–15]). Male lures have two main roles in management programs. First, the lures are commonly used in detection trapping programs to identify incipient infestations [16]. In addition, male lures are used in the Male Annihilation Technique (MAT) to suppress or eradicate invasive populations [17].

Operationally, MAT involves the distribution in the infested area of large numbers of dispensers impregnated with a male lure and a toxicant in order to reduce male abundance to such a low level that population suppression or eradication results. Although MAT may be used alone, it is often combined with other control methods, such as the sterile insect releases and/or protein bait sprays. The use of a highly attractive male lure is critical to MAT’s effectiveness, and historically it has been most successfully used against ME-responding males and, in particular, the oriental fruit fly, *Bactrocera dorsalis* (Hendel). In several well-known cases, the implementation of ME-based MAT, either alone or with other control tactics, has resulted in the completion extirpation of island populations of *B*. *dorsalis* populations [18–21].

In their review of MAT, Vargas et al. [17] list 10 programs, mostly in tropical Asia and Oceania, that used MAT in control efforts against *B*. *dorsalis* and document great variation in both the particular materials and procedures used in these different MAT operations. For example, different materials served as ME-dispensers, with cane-fiber boards [19], coconut husks [22], and cotton rope [20], among others, being used in different locations. In addition, deployment of ME-laden dispensers was accomplished by ground placement [22,23], aerial drop [18,24], or both ground and aerial application [19,20]. Finally, and perhaps most importantly, there was large variation in the amount of ME applied to individual dispensers (e.g., 8–23 g [19,20]) and the density at which individual ME-dispensers were distributed in the environment (e.g., 85–400 dispensers/km^2^ [18,22]. In extreme cases, about 5000 dispensers/km^2^ were deployed [25]), and the total dose of ME applied per unit area was (2–22 kg total ME/km^2^ [18,24]).

MAT has also been adopted to eradicate localized outbreaks of ME-responding species in otherwise fruit fly free areas. In California, for example, a grid of ME-baited and food-based traps operates continuously over the Los Angeles basin and surrounding area [26]. Discovery of an inseminated *B*. *dorsalis* female, repeated finds of fertile males in short time period, or larval-infested fruit may trigger an eradication effort. As part of the eradication program, a waxy paste formulation, SPLAT-MAT-ME with spinosad, is spot-applied (5 grams per spot) to utility poles and tree trunks at a minimum density of 230 sites per km^2^ (600 per mile^2^) within a 2.4 km (1.5 mile) radius around the detection location [27]. SPLAT (Specialized Pheromone and Lure Application Technology) is a proprietary formulation of biologically inert materials that allows controlled release of volatile compounds (such as ME) with or without accompanying pesticides [28,29] Spinosad is a natural pesticide derived from fermentation products of the soil bacterium *Saccharopolyspora spinosa* Mertz and Yao found to be effective for long intervals against tephritid fruit flies [30]. In California, SPLAT-MAT-ME is applied every 2 weeks in the target area until eradication is declared (i.e., an interval equivalent to three generations of *B*. *dorsalis* elapsed without further detection).

While the MAT protocol against ME-responding *Bactrocera* species has been highly effective, the associated cost is quite high. In California alone, on average, about 5 such MAT projects have been performed per year over the past 4 years, with each project costing approximately $200,000 (J. Leathers, Pers. Comm.). The present study expands upon earlier, less comprehensive field tests [31,32] investigating the notion that a lower density of SPLAT-MAT-ME sites might actually that be more effective in attracting (and eliminating) *B*. *dorsalis* males than the site density currently used in programs around the world. These earlier studies suggested, counterintuitively, that “less is better” as olfactory interference (or competition) resulting from a high density of ME sources may inhibit male ability to locate individual point sources (as shown for pheromone-baited traps and moth captures, e.g., [33–35]). Here, we made direct comparisons of trap captures of marked *B*. *dorsalis* males released under three experimental SPLAT-MAT-ME site densities, i.e., 110, 220, and 440 per km^2^. In addition, trap effectiveness was compared among these different densities for both fresh and aged traps to evaluate whether weathering of the SPLAT-MAT-ME formulation influenced any density effects observed.

## Materials and methods

### Study site

Field work was conducted in a macadamia nut orchard (*Macadamia integrifolia* Maiden & Betche) covering 445 ha (elevation 170 m) in Keaau on the windward coast of Hawaii Island (commonly known as the Big Island), Hawaii. We received permission from the land owner to conduct our study in the orchard, which did not involve endangered or protected species. Macadamia is neither an ovipositional nor adult food source, thus eliminating these parameters as potential influences on the distribution of released flies. Trees were of uniform size, with height of approximately 5 m and ground canopy cover of approximately 30 m^2^. Tree rows were 8–9 m apart, and within a row trees were spaced at 4–5 m intervals (trunk-to-trunk). Field work was conducted during two time periods in 2017, namely April-June and August-October. Average daily temperatures were similar between these periods, i.e., 22.1°C and 22.9°C for April-June and August-October, respectively. Total rainfall was 60.5 cm for the initial period and 92.0 cm for the second period. Weather data were obtained from a NOAA-operated weather station in Hilo, HI, 6 km from the study site (19^o^ 38’ 34.30” N, 155^o^ 4’48.13” W).

Three rectangular plots were established within the orchard, each with approximate dimensions of 0.9 x 0.6 km and an area of 0.51 km^2^ (exact location given in Table 1). The minimum distance between plots was 0.25 km, and the minimum distance between release transects (described below) was 0.68 km. Each plot contained 56 rows of trees with approximately 230 trees per row. To reduce entry by wild flies into the study plots (and correspondingly the time spent counting captured, marked-and-released flies within the study plots), a ring of about 100 ME-baited bucket traps was established around the perimeter of the entire study area four weeks before the start of releases and maintained continuously during each of the 3-month study intervals. The minimum distance between bucket traps and each of the experimental plots varied between 35 m and 135m. Bucket traps are fully described in [36] and briefly are 5 L in volume with four entrance holes evenly spaced around the side and four drain holes on the bottom. A 10 g ME plug plus a kill strip (2,2-dichlorovinyl dimethyl phosphate [DDVP]; Vaportape II, Hercon Environmental, Emigsville, PA) was placed in each bucket trap. Captured and killed flies were removed regularly, and the bait and kill strip were replaced every 12 weeks.

**Table 1:** Locations of experimental plots. Each plot had an area of 0.51 km^2^. Control releases were conducted in an area centered at N 19°36.913, W 155°05.339, but no grid applies.

Plot	Corner 1	Corner 2	Corner 3	Corner 4
A	N 19°36.491; W 155°04.765	N 19°36.626; W 155°04.291	N 19°36.751; W 155°04.902	N 19°36.893; W 155°04.392
B	N 19°37.179; W 155°04.956	N 19°36.904; W 155°04.837	N 19°37.034; W 155°04.441	N 19°37.306; W 155°04.533
C	N 19°36.540; W 155°05.568	N 19°36.256; W 155°05.423	N 19°36.399; W 155°04.971	N 19°36.683; W 155°05.077

### Insects

Released flies were obtained from a bisexual colony produced at the USDA-ARS Daniel K. Inouye Pacific Basin Agricultural Research Center, Hilo, HI. This colony was started in 1991 (approximately 312 generations under domestication) and has been reared following standard protocol [37]. The colony is housed in a building devoted exclusively to rearing, which is maintained at 22.5 ± 1°C, 55% ± 3% RH, and a 14:10 L:D photoperiod.

Before placing pupae in adult eclosion boxes, they were marked using fluorescent dye following the standard procedure in SIT programs [38]. As described below, releases were made concurrently in the three study plots. The flies released in the different plots were marked with different colors, thus allowing assessment of potential inter-plot dispersal. Upon emergence, the flies generally retain dye particles on the body that can be viewed with a dissecting microscope under UV (black light). The head of each captured fly was crushed with the blunt end of a dental instrument dipped in acetone against filter paper, such that dye particles caught in the ptilinum during pupal eclosion dissolved in the acetone and coloration was visible under UV. Dye colors used in marking flies included horizon blue, arc yellow, Saturn yellow, and fire orange (DayGlo Corporation, Cleveland, Ohio, US), and each color was applied at a dose of 2 g per L of pupae.

A sample of non-dyed pupae was taken from each production batch used for the releases and used in standard quality control tests, measuring pupa-to-adult emergence rate and adult flight ability (following [39]). One emergence grid (holding 100 pupae) and two flight tubes (each with 100 pupae) were monitored per production batch. Additionally, a small number of males were tested for ME responsiveness immediately following release of each cohort [9]. Briefly, 15 sexually mature males (12 d old) were released in a glass Y-tube olfactometer and monitored for response to methyl eugenol versus no odor (blank control). Treatments were switched between arms of the olfactometer, and the test was repeated with another set of 15 males (i.e., 30 males total tested per release). These quality control parameters were used to estimate the number of flight-capable and responsive adults as a fraction of the pupal volume.

To obtain flies for release, 100 mL of non-irradiated, dyed pupae (approximately 5,000 flies) were placed in individual PARC boxes 2 d prior to emergence. These containers, which until recently were the type routinely used in SIT programs, are opaque, plastic boxes (0.48 by 0.60 by 0.33 m) that contain mesh screening on the sides and the top for ventilation [40]. A granular mixture of sugar and protein yeast hydrolysate (3:1 v:v) was placed, as a circular cake (6 cm diameter, 2 cm thick), on the top screen through which the flies could feed. An agar block (15 by 10 cm, 5 cm thickness) was also provided as a water source. Both food and water were replaced after 7 d. The holding boxes were kept under the same environmental conditions as the colony.

### Preparation, deployment, and density of SPLAT sites

The same SPLAT-MAT-ME with spinosad formulation as used in California was used in the present study. A large syringe was used to apply 8 mL (51% ME, 2% spinosad [a mixture of spinosyn A and spinosyn D]) of the formulation to individual wooden blocks (10 by 8 by 0.5 cm thick); hereafter, the wooden blocks holding the SPLAT, ME, and spinosad mixture are termed SPLAT sites, and the set of SPLAT sites comprise a MAT grid. In the study plots, SPLAT sites were deployed in large plastic delta traps (LPD traps) or in Jackson traps (Scentry Biologicals, Inc., Billings, MT). The LPD traps contained sticky inserts to capture flies and served as monitoring devices of the attraction of released flies. For each replicate in each study plot, the same number (N = 55) of LPD traps was deployed evenly as part of the MAT grid. In contrast, Jackson traps held SPLAT sites but lacked sticky inserts and hence did not provide data on fly captures. In fact, floors of the Jackson traps were cut length-wise and opened to prevent the build-up of dead flies, which may have blocked access to the lure. The function of these modified Jackson traps was to simulate varying MAT grid densities, creating “olfactory” environments with varying numbers of ME sources per unit area. To emphasize this point and avoid possible confusion with the LPD devices, which actually did function as traps, we hereafter refer to the modified Jackson traps as “Jackson hats”, since the body of the device served only as a cover of the contained SPLAT site.

We experimentally established low, high, and super high densities of SPLAT sites by deploying (along with the LPD traps) 0, 55, or 165 Jackson hats in the study plots. Thus, the total numbers of SPLAT sites were 55 for the low (55 LPD, 0 Jackson hats), 110 for the high (55 LPD, 55 Jackson hats), and 220 for the super high (55 LPD, 165 Jackson hats) density treatments. These numbers correspond to SPLAT site densities of 110/km^2^, 220/km^2^ (similar to the minimum MAT site density used in CA), and 440/km^2^ for the three experimental treatments, respectively. Regarding their specific placement, in all treatments LPD traps were placed in every 5^th^ row between rows 5 and 50, i.e., away from the edges of the plot, with either 5 or 6 traps per row. For the high and super high treatments, Jackson hats were placed evenly between LPD traps in a given row.

### Release-recapture protocol

As noted above, fly releases were performed simultaneously in the three experimental plots, each assigned to a particular density of SPLAT sites. Following completion of a release-recapture cycle (described below in detail), the SPLAT site density treatments were rotated among experimental plots, such that within both the April-June and August-October study intervals each SPLAT site treatment was established within each of the three plots. Successive cycles were separated by 1–2 weeks within each study period. Thus, for each SPLAT site density, fly captures were monitored for 6 total replicates (3 plots/study interval, 2 study intervals).

Release-recapture cycles followed the same protocol and schedule in all experimental plots over the entire study. The LPD traps and Jackson hats were prepared and deployed in the field 1 d before fly release. Flies were released from the back of a truck driven slowly (5–10 km/h) along a 320 m transect in the center of the plot. Releases were made at 1000 hrs by opening the PARC boxes and striking them to promote flight. A leaf blower was used to disperse flies reluctant to leave the box or on the truck bed. Of the approximately 6,300 males and equal numbers of females brought to each plot, an estimated average flight-capable and ME responsive number of 4470 males and 5065 flight-capable females were released per plot from two PARC boxes; flies were 10 d old and sexually mature [41,42]. Mortality in the holding boxes was not quantified but was minimal (estimated 1–3%). Sticky inserts from the LPD traps were removed 1 and 4 d after a release, and the captured flies were returned to the laboratory for identification and counting. Inserts were replaced at the 1 d post-release check but not at the 4 d post-release. The LPD and Jackson hats were left in the field for weathering, and two weeks after the first release, the procedure described above was repeated. For the control plot the same procedure was followed except no SPLAT sites were deployed.

### Female recaptures

To assess impact of MAT on female flies [32] and to compare capture without ME between cohorts, each MAT grid also included six torula yeast-baited McPhail (multilure; Better World Manufacturing) traps evenly placed 100 meters on either side of the release transect (three pairs). Each trap contained 300 ml of torula yeast solution and was checked for male and female trap captures five days after each fly release. We also set six torula yeast baited McPhail traps following the same protocol as above in a fourth release area with no SPLAT sites of any type, where we also conducted releases (hereafter the “control plot”).

## Results

The numbers of marked male *B*. *dorsalis* recaptured at the LPD traps for the 1d and 14 d old MAT grids and in the protein traps are given in Tables 2 and 3, respectively. The estimated male kill is also shown, calculated by multiplying the number captured in the LPD traps by 2 or 4 (for 220 and 440 spots/km^2^) to account for kills by the Jackson hats.

**Table 2:** Release, recapture and quality control data for fresh (1d old) replicates. Estimated number released in each plot based on pupal volume and an even sex ratio was 6,300 each of males and females. Considering the emergence proportion, proportion responders to ME, and proportion of fliers, the estimated number of males available for capture per plot varied between 3,427 and 4,957 across trials.

Release Date	Treatment*	Plot	Number of recaptured males	Estimated number of males killed	Emergence proportion	Proportion fliers	Proportion males responding to ME	Number of females captured in protein traps	Number of males captured in protein traps
12-Apr-2017	110	A	1746	1746	0.895	0.855	0.850	2	0
	220	B	826	1652				0	0
	440	C	393	1572				1	0
	0	-	-	-				2	0
3-May-2017	110	C	1669	1669	0.900	0.880	0.733	4	0
	220	A	746	1492				3	0
	440	B	447	1788				3	0
	0	-	-	-				5	0
31-May-2017	110	B	1951	1951	0.905	0.820	0.733	6	0
	220	C	613	1226				6	0
	440	A	300	1200				7	1
	0	-	-	-				9	6
1-Aug-2017	110	A	1594	1594	0.980	0.860	0.933	9	1
	220	B	707	1414				11	0
	440	C	192	768				17	0
	0	-	-	-				33	0
23-Aug-2017	110	C	1372	1372	0.970	0.840	0.967	17	0
	220	A	806	1612				9	0
	440	B	323	1292				8	1
	0	-	-	-				10	3
13-Sep-2017	110	B	1732	1732	0.930	0.875	0.967	5	1
	220	C	541	1082				15	4
	440	A	287	1148				7	0
	0	-	-	-				4	5

* spots/km^2^.

**Table 3:** Release, recapture and quality control data for aged (14d old) replicates. Estimated number released in each plot based on pupal volume and an even sex ratio was 6,300 each of males and females. Considering the emergence proportion, proportion responders to ME, and proportion of fliers, the estimated number of males available for capture per plot varied between 4,488 and 4,797 across trials.

Release Date	Treatment*	Plot	Number of recaptured males	Estimated number of males killed	Emergence proportion	Proportion fliers	Proportion males responding to ME	Number of females captured in protein traps	Number of males captured in protein traps
26-Apr-2017	110	A	1664	1664	0.905	0.87	0.967	2	0
	220	B	773	1546				2	0
	440	C	194	776				2	1
	0	-	-	-				10	9
17-May-2017	110	C	1941	1941	0.895	0.855	0.967	6	1
	220	A	833	1666				5	1
	440	B	343	1372				0	0
	0	-	-	-				4	6
14-Jun-2017	110	B	2365	2365	0.925	0.855	0.933	10	2
	220	C	882	1764				28	1
	440	A	474	1896				24	6
	0	-	-	-				25	6
16-Aug-2017	110	A	3331	3331	0.99	0.83	0.867	14	2
	220	B	1116	2232				10	0
	440	C	588	2352				32	8
	0	-	-	-				3	2
06-Sep-2017	110	C	1474	1474	0.99	0.86	0.867	12	1
	220	A	1056	2112				21	1
	440	B	292	1168				7	1
	0	-	-	-				7	1
27-Sep-2017	110	B	2022	2022	0.99	0.87	0.8	11	1
	220	C	650	1300				37	1
	440	A	483	1932				24	0
	0	-	-	-				6	0

* spots/km^2^.

**Fig 1:**
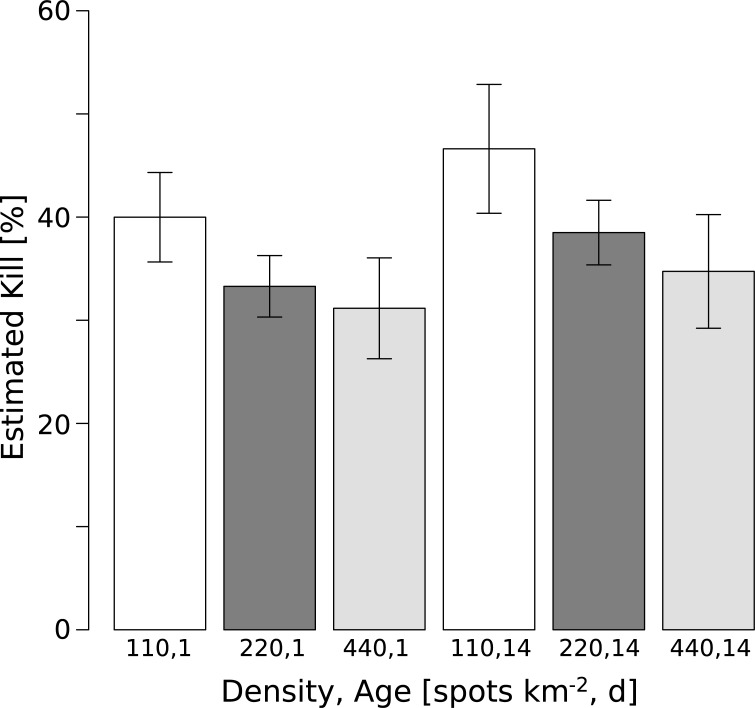
Mean and SE of estimated percent males killed for each application density and grid age. Percentages were calculated via dividing the estimated male kill by the product of the number of pupae per release, proportion emergence, proportion flight ability, proportion ME responders, and 0.5 (assuming an even sex ratio).

The average percentage of males killed per combination of application density and grid age are shown in Fig 1. Decreasing percentage of males killed is seen with increasing application density for releases at 1d and 14d. Slightly higher average kill percentage for any given density is apparent for releases in the weathered grids, but the variance is also somewhat higher in those instances.

An ANOVA on the estimate number killed indicates a statistically significant difference in the estimated number of males captured across application densities and the two age levels, but no significant interaction (Table 4). A Tukey HSD test showed a significant difference between 110 and 440 spots/km^2^ (*p* = 0.020), but not between 110 and 220 (*p* = 0.260) or 220 and 440 (*p* = 0.430).

**Table 4:** ANOVA of log(number estimated killed) as predicted by application density, grid age, and their interaction. Log transformed response variable was used to ensure homogeneity of variances as assessed via Bartlett’s test.

Factor	df	SS	MS	*F*	*p*
Density	2	0.567	0.284	4.112	0.027
Age	1	0.360	0.360	5.226	0.030
Density*Age	2	0.004	0.002	0.029	0.972
Residuals	30	2.069	0.070		

**Fig 2:**
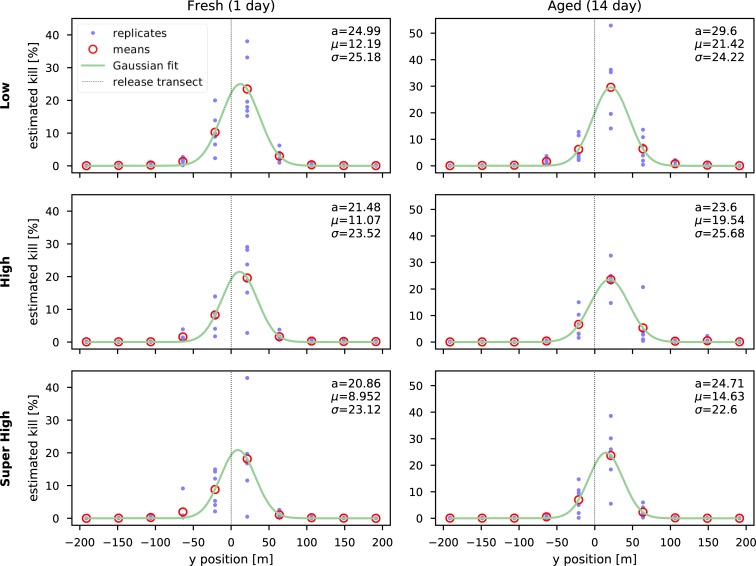
Relationship between distance from release transect on estimated percentage of males killed. Each panel is a combination of density treatment (rows) and MAT age (columns). For each replicate, the total estimated percentage of the males killed (including male kills for hats) for each row of traps is shown with a blue dot. The x axis is the position of the rows relative to the release transect. The mean for all six replicates at each trapping row is shown by a red circle. A Gaussian function was fitted to the data and is shown by the green line and the fit parameters given in the upper left of each panel.

The pattern of recaptures based on distance from the release transects was similar across experimental treatments (Fig 2). For the fresh grids, low density had an average 86.1% recaptures in the nearest two rows to the release transect. For the high and super high fresh grids the values were 86.9% and 86.4%. For the aged grids we observed slightly more variation in this measure: 78.1% for low density, 83.4% for high, and 90.8% for super high density. Further details on the spatial pattern of recaptures is given in the supporting information file (S1 File), which also includes a more sophisticated method of estimating overall kill (interpolation) yielding the same qualitative results as above. The SI also includes visualization of trap catches with the enhanced interpolation.

Recaptures in McPhail traps were heavily female biased (Tables 2 and 3). Analysis of female recaptures via a second ANOVA tested the effect of application density, MAT grid age, plot location, and their full interaction. It did not show any significant effects (*p* > 0.05). The response (number of females) was transformed using log(1+*x*) to conform to the assumption of normality as tested via the Shapiro-Wilk procedure.

## Discussion

With the results of this study there is now increasing evidence supporting the hypothesis that the effectiveness of MAT with a powerful male lure, such as ME, is reduced when application density exceeds a relatively low threshold [31,32]. In the current study, the lowest application density (110 spots/km^2^) was the most effective as measured by estimated males killed, leaving the possibility that an even lower density might further increase effectiveness. The mechanism responsible for reduced catch in the higher application densities is not resolved, but the most likely hypothesis is interference: a large amount of lure in the air reduces the ability of individual males to follow odor plumes or gradients to point sources. This has been termed the “MAT-ME saturation hypothesis”, effected by the same principle at work in trap interference [43,44] and used for pest control via mating disruption (e.g. [45]).

Failure to arrive at point sources means that males can’t ingest the insecticide, making MAT ineffective. Spatial patterns of recapture in our current data did not reveal any differences in the locations of captures relative to the release transect when controlling for the overall number recaptured (Fig 2), which does not support interference as the main mechanism. However, while the spatial patterns were similar, the overall catch was significantly lower at the higher application densities, and interference remains a viable hypothesis. Tests aimed specifically at this question would be better suited to examine the mechanistic basis of the lower catch in the higher density grids.

It is also noteworthy that the aged MAT grids were significantly more lethal to male *B*. *dorsalis*, suggesting that reapplication intervals should also be examined. For tephritids it is generally accepted that weathered lures are less attractive than fresh [46–49], but there are cases where aged lures are more attractive (i.e., capture more flies per unit time). Working with *B*. *dorsalis* and MAT-ME, Vargas et al [50] reported higher catches with two or four week old lures compared with those aged one week. Various explanations besides saturation might account for these observations, including receptor overloading [51] and, in the case where an insecticide is used with the lure, repellence or mortality before the insect enters a trap [52]. Changes in reapplication intervals must also consider toxicant durability.

Manoukis et al [32] found a decrease in estimated female survivorship in a field experiment on MAT exploring the effect of spot application density, a result that has also been occasionally reported in other field and laboratory studies [19,53,54]. Our results from the McPhail trap captures did not show a difference in female capture between the control and the various tested MAT application densities, but the generally low number of recaptures in the protein traps limits the power of any analysis to detect such a difference. Further experiments on this question may be warranted.

The spatial distribution of MAT spots in real-world programs can be uneven, in contrast to the pattern used for this study. Placement along public rights-of-way, for example, would lead to an irregular grid pattern. Further, if some areas are inaccessible, there may be surplus spots applied to accessible portions of subunit within a layout- this could lead to higher density in the treated area reducing effectiveness there.

Two other differences between this experiment and real-world situations are worth highlighting: 1) we used only colony-reared *B*. *dorsalis* and 2) weather/climatic and other environmental conditions may differ between our study site and other locations. Mass-reared tephritids are known to vary from wild counterparts in various aspects including development, sexual competitiveness, fertility, and survivorship [55–57]. However, since colony-reared individuals were used in all comparisons we don’t expect these factors to lead to important differences compared with wild flies, barring any qualitative difference in lure response. Studies to date show a similar response from wild and colony flies to ME, though responsiveness increases later for the former due to their longer sexual maturation time [53]. Weather conditions can certainly affect lure weathering and effectiveness over time, but these results should hold for relatively fresh grids.

Environmentally, this experiment was conducted in a non-host orchard, which may lead to quantitatively different results than if host fruit were available due to differences in the olfactory environment. Background odors can affect insect behavior [58], and so it is possible that lure responsiveness could be increased or decreased by presence of host fruit [59]. Host odors could also mask lures, decreasing the ability of males to find MAT spots e.g. [60]. To our knowledge no data exist on attractiveness of ME in habitats with hosts versus non-hosts, so the impacts on this study’s results are unknown. Another possibility is that male movement might be influenced by the presence of host plants, perhaps affecting probability of death by MAT [61].

Another open question is whether the reduced effectiveness of higher application densities is seen in MAT against other species, for example cue-lure based MAT to control *Zeugodacus cucurbitae* or *Bactrocera tryoni* [62–64]. In general cuelure is considered to be less attractive to responding species than ME to its responders [8,9]. If attraction is driven by sensitivity to the odor of the lure, then perhaps less interference might be expected for cuelure based MAT compared with ME.

Clear benefits for control and eradication programs would be attained from reducing the application density of MAT against *B*. *dorsalis*. These include a reduction of 50% in the material applied and lower labor costs. Beyond cost savings, this study supports previous findings [31,32] that lower densities are more effective for ME-based MAT, and application of these results should improve the safeguards against this highly invasive agricultural pest. Additional research in areas where MAT programs are currently undertaken would be helpful to corroborate this study’s findings.

## Supporting information

S1 File Visual representation of all LPD recaptures (blue) and interpolated hat kill estimates (red). Grey × mark LPD and hat locations. The area of each circle corresponds to the total number of flies caught or estimated killed at that location. The treatment, date, total number recaptured (Σ*trap*), and total estimated killed (Σ*interp*) is given in the title of each subplot. Each figure covers a single full replicate.
(PDF)
