# Supplementary material for: A field test on the effectiveness of male annihilation technique against Bactrocera dorsalis (Diptera: Tephritidae) at varying application densities
Source: PLoS One. 2019 Mar 8;14(3):e0213337. doi: 10.1371/journal.pone.0213337 (PMC6407772; doi:10.1371/journal.pone.0213337)

Supporting Information (S1 File)

A Field Test on the Effectiveness of Male Annihilation Technique against *Bactrocera dorsalis* (Diptera: Tephritidae) at Varying Application Densities

The simple method (multiplication) of estimating the number of flies killed by unmonitored hats presented in the paper assumes that there is no difference in relative distance of “Jackson hats” and large plastic delta traps (LPD traps) from the release point of each fly. The trapping grid was laid out as uniformly as possible and flies released along a transect to make this a reasonable assumption in the aggregate, but vagaries of release and field conditions means it isn't perfect. We conducted an alternative analysis estimating hat kills by linear interpolation of the LPD catches along each row (parallel with the release transect). We believe the results of this approach are slightly more accurate than the simple method presented in the main paper, but since the interpolation approach produces lower kill estimates, the simple approach is a more conservative test of our main results.

Table A gives the estimated kills from the simple multiplication method (est. kill) alongside those produced by row-wise interpolation (interp. kill). Figures A through F show the LPD catches (blue) and interpolated hat kill estimates (red) for every trap servicing (2 services per treatment × age × replicate).

Performing the same statistical analysis procedure presented in the main paper on the log transformed interpolated kill estimates results in extremely similar results. The ANOVA of interpolated number killed shows significant effect of treatment ($p=0.010$) and MAT age ($p=0.026$) with no significant interaction. A Tukey HSD test against shows significance when comparing 110 and 440 spots per km ($p=0.0076$; the simple estimation method gave $p=0.020$), but was not significant for 110 vs 220 ($p=0.186$) or 220 vs 440 ($p=0.325$).

Table A: Comparing the number of flies recapture by LPD, estimated kills (est. kill) using the simple multiplication method used in the main paper, and row-wise liner interpolation (interp. kill). See Table 2 and Table 3 in the main paper for additional information.

SPLAT age	date	treatment	recaptured	est. kill	interp. kill
01d	4/12/2017	110	1746	1746	1746
		220	826	1652	1616
		440	393	1572	1430
	5/3/2017	110	1669	1669	1669
		220	746	1492	1447
		440	447	1788	1755
	5/31/2017	110	1951	1951	1951
		220	613	1226	1195
		440	300	1200	1152
	8/1/2017	110	1594	1594	1594
		220	707	1414	1363
		440	192	768	741
	8/23/2017	110	1372	1372	1372
		220	806	1612	1508
		440	323	1292	1221
	9/13/2017	110	1732	1732	1732
		220	541	1082	1079
		440	287	1148	1100
14d	4/26/2017	110	1664	1664	1664
		220	773	1546	1531
		440	194	776	739
	5/17/2017	110	1941	1941	1941
		220	833	1666	1625
		440	343	1372	1349
	6/14/2017	110	2365	2365	2365
		220	882	1764	1736
		440	474	1896	1835
	8/16/2017	110	3331	3331	3331
		220	1116	2232	2251
		440	588	2352	2271
	9/27/2017	110	2022	2022	2022
		220	650	1300	1272
		440	483	1932	1887
	9/6/2017	110	1474	1474	1474
		220	1056	2112	2050
		440	292	1168	1048

Figures A through F: (on following pages)
Visual representation of all LPD recaptures (blue) and interpolated hat kill estimates (red). Grey × mark LPD and hat locations. The area of each circle corresponds to the total number of flies caught or estimated killed at that location. The treatment, date, total number recaptured (Σ_{trap}), and total estimated killed (Σ_{interp}) is given in the title of each subplot.
Each figure (A-F) covers a single full replicate.

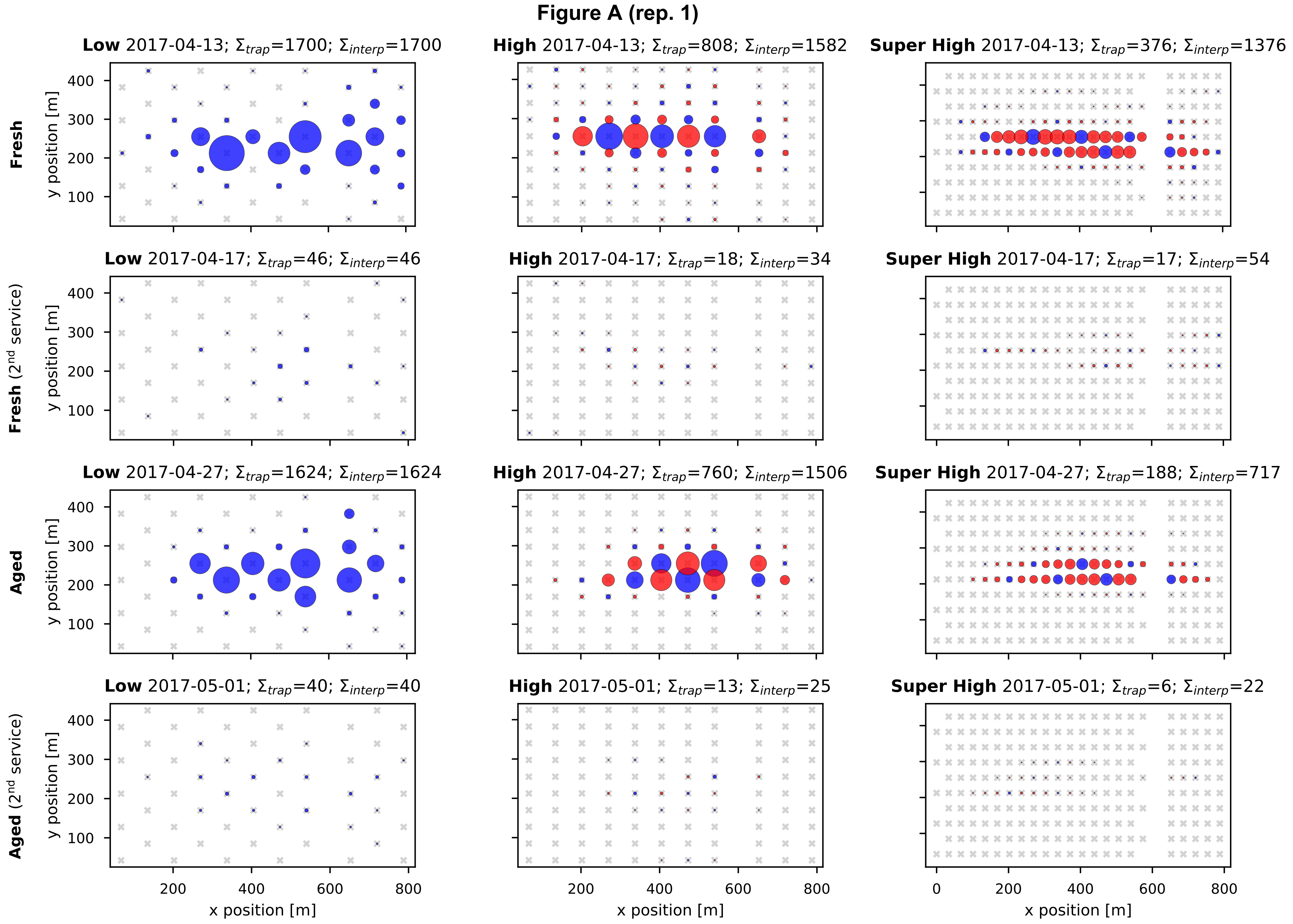

Figure A (rep. 1)
Low 2017-04-13; Σtrap=1700; Σinterp=1700
High 2017-04-13; Σtrap=808; Σinterp=1582
Super High 2017-04-13; Σtrap=376; Σinterp=1376
Low 2017-04-17; Σtrap=46; Σinterp=46
High 2017-04-17; Σtrap=18; Σinterp=34
Super High 2017-04-17; Σtrap=17; Σinterp=54
Low 2017-04-27; Σtrap=1624; Σinterp=1624
High 2017-04-27; Σtrap=760; Σinterp=1506
Super High 2017-04-27; Σtrap=188; Σinterp=717
Low 2017-05-01; Σtrap=40; Σinterp=40
High 2017-05-01; Σtrap=13; Σinterp=25
Super High 2017-05-01; Σtrap=6; Σinterp=22
Fresh
Fresh (2nd service)
Aged
Aged (2nd service)
y position [m]
x position [m]
100
200
300
400
0
200
400
600
800

Figure B (rep. 2)

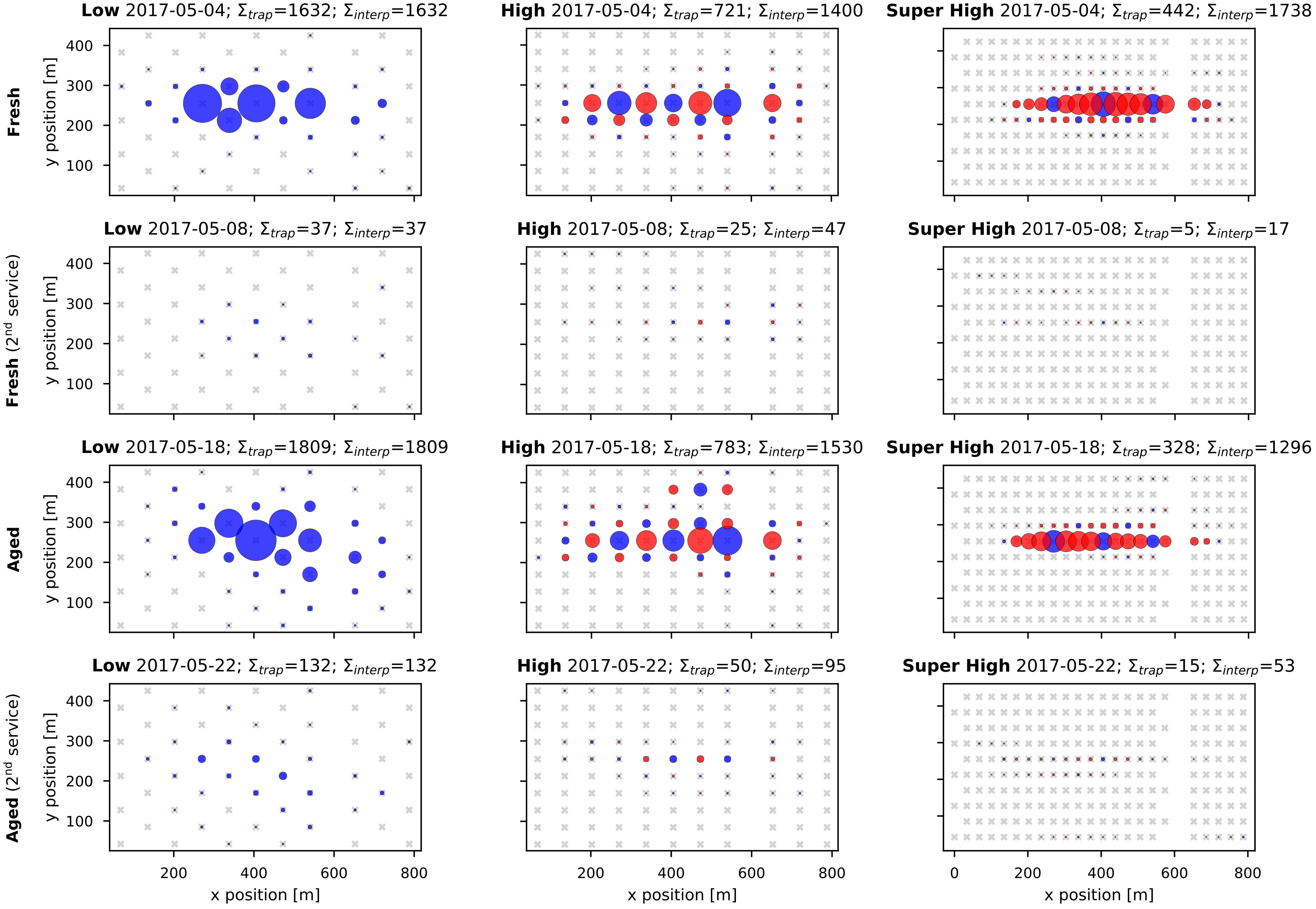

Figure C (rep. 3)

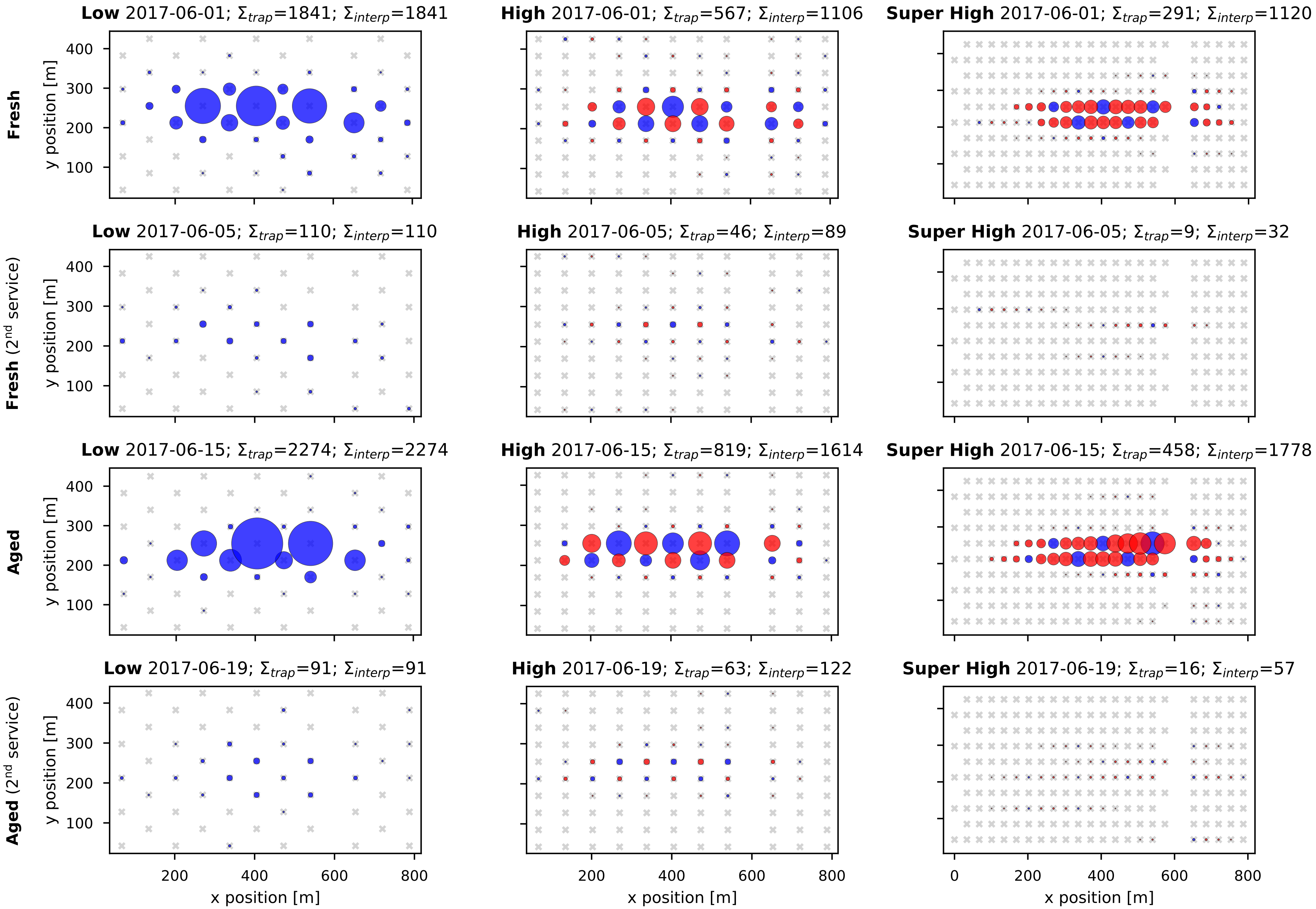

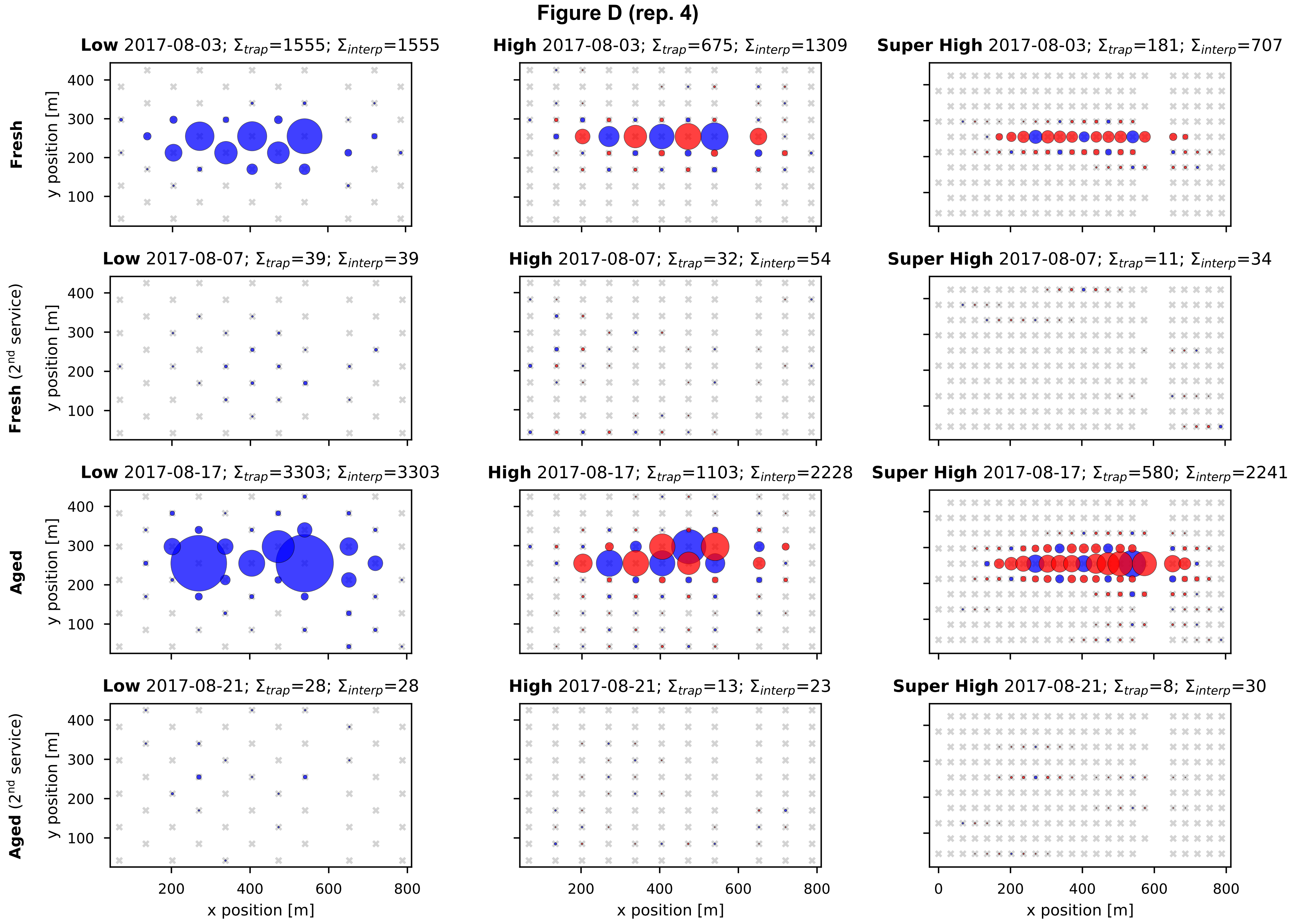

Figure D (rep. 4)
Low 2017-08-03; Σtrap=1555; Σinterp=1555
High 2017-08-03; Σtrap=675; Σinterp=1309
Super High 2017-08-03; Σtrap=181; Σinterp=707
Low 2017-08-07; Σtrap=39; Σinterp=39
High 2017-08-07; Σtrap=32; Σinterp=54
Super High 2017-08-07; Σtrap=11; Σinterp=34
Low 2017-08-17; Σtrap=3303; Σinterp=3303
High 2017-08-17; Σtrap=1103; Σinterp=2228
Super High 2017-08-17; Σtrap=580; Σinterp=2241
Low 2017-08-21; Σtrap=28; Σinterp=28
High 2017-08-21; Σtrap=13; Σinterp=23
Super High 2017-08-21; Σtrap=8; Σinterp=30
Fresh
Fresh (2nd service)
Aged
Aged (2nd service)
y position [m]
x position [m]

Figure E (rep. 5)

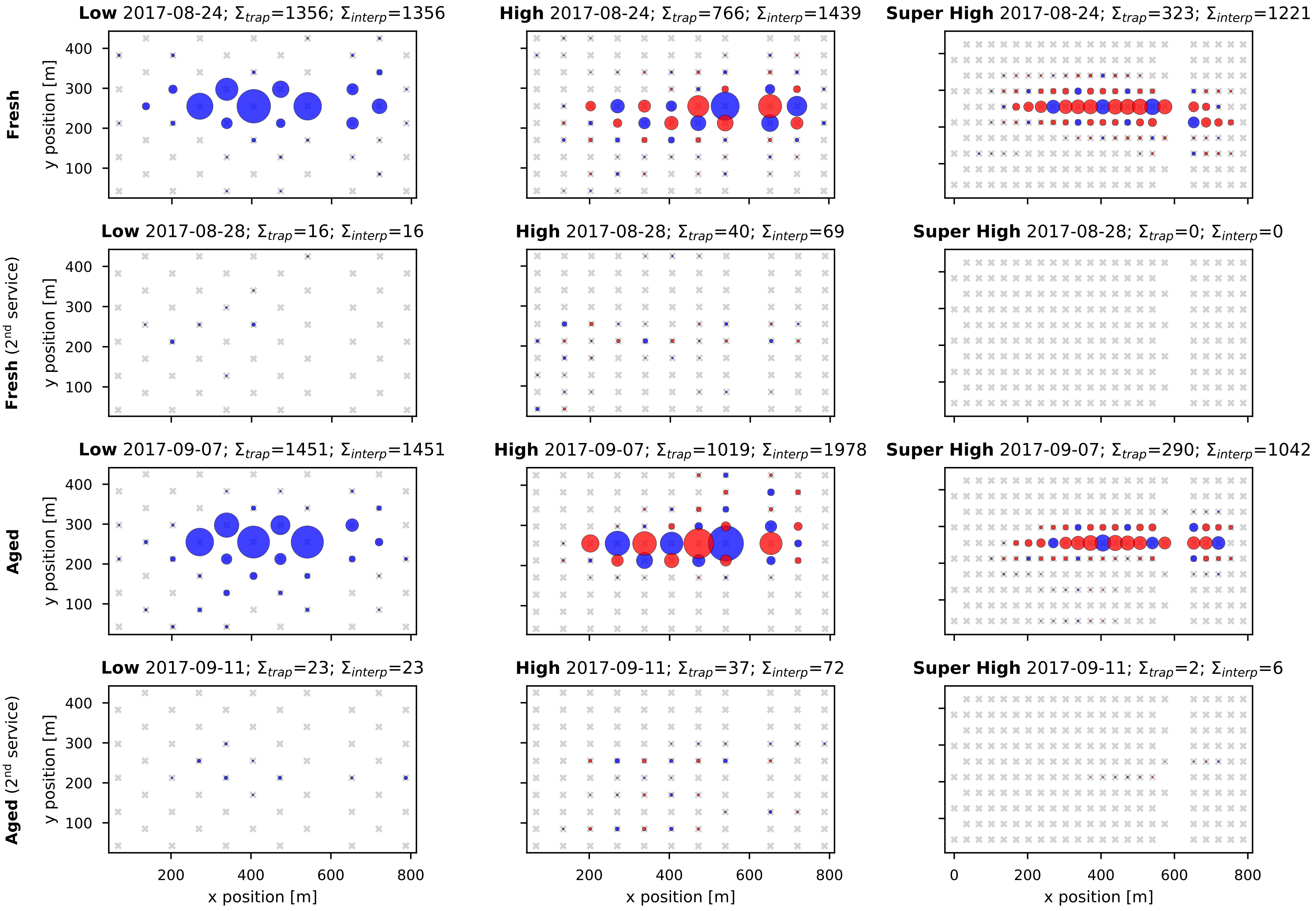

Figure F (rep. 6)

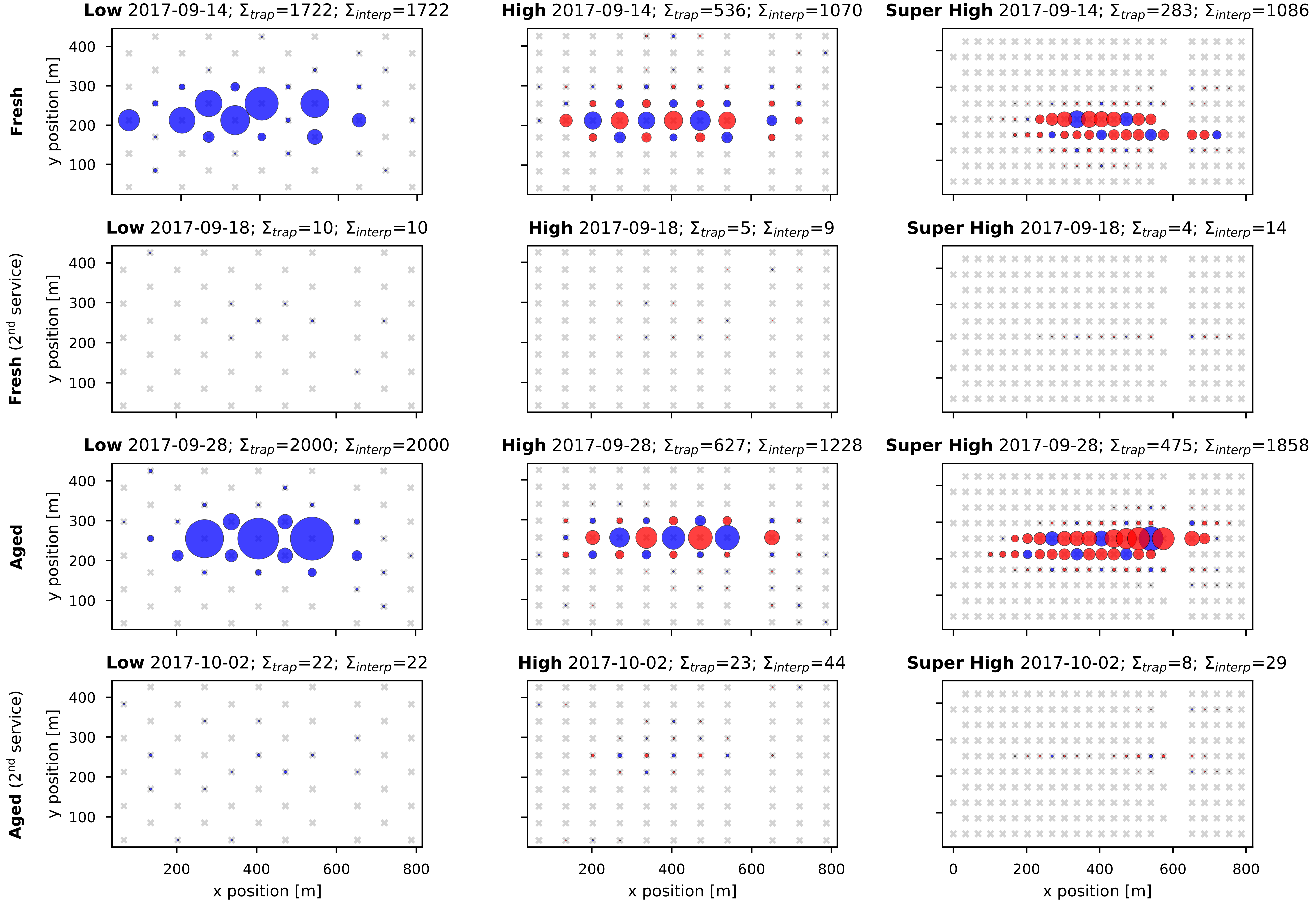

Supplement: S1 File — Grey × mark LPD and hat locations. The area of each circle corresponds to the total number of flies caught or estimated killed at that location. The treatment, date, total number recaptured (Σtrap), and total estimated killed (Σinterp) is given in the title of each subplot. Each figure covers a single full replicate. (PDF) [file pone.0213337.s001.pdf]
